# Remarks on the Medico-Legal Aspect of Intestinal Surgery

**Published:** 1892-03

**Authors:** W. E. B. Davis

**Affiliations:** Rome, Ga.; Formerly Surgeon to the Birmingham Hospital of United Charities; President of the Tri-State Medical Society of Alabama, Georgia, and Tennessee; Secretary of the Southern Surgical and Gynecological Association, etc., etc.


THE
### Southern Medical Record.
A MONTHLY JOURNAL OF MEDICINE AND SURGERY.

Vol. XXII. Atlanta, Ga., March, 1892. No. 3

### Original Articles.

### REMARKS ON THE MEDICO-LEGAL ASPECT OF INTESTINAL SURGERY.

(A discussion of Dr. J. D. S. Davis' paper, at the Fourth Annual Meeting of the S. S. & G. Association.)

BY W. E. B. DAVIS, M. D., ROME, GA.

Formerly Surgeon to the Birmingham Hospital of United Charities; President of the Tri-State Medical Society of Alabama, Georgia, and Tennessee; Secretary of the Southern Surgical and Gynecological Association, etc., etc.

There are some embarrassing points in reference to the matter under discussion. It is embarrassing for an expert witness to testify in a case where a surgeon has been operating and left the intestines out for an hour and the patient dies. I have had some experience in hearing witnesses testify in such a case. It is not an easy matter for an expert witness to testify in a case where he wants to protect the surgeon and where the latter does not deserve to be protected.

While Dr. Senn has done a great work in intestinal surgery, I do not consider his gas test as valuable a means of diagnosis as a great many think. It may reveal a perforation, but if it should not, it would be a great risk to rely upon it, without opening the abdomen. Another thing; when the gas is used to distend the intestines we increase the shock, and get a condition which is difficult to relieve ; hence, the gas test is altogether unsatisfactory. Even if it could be relied on to show whether the intestine has been injured or not, we may have other viscera injured, and we would let our patient die. An exploratory incision is not dangerous in careful hands, and there will then be an opportunity of arresting hemorrhage.

The recent observations of a number of surgeons show that hemorrhage is more often the cause of death in these cases than we had been led to believe. The intestine is not perforated as often as is taught in the books.

In reference to delay in these operations, it is very dangerous to call a general practitioner into consultation in penetrating wounds of the abdomen. He will caution delay. He will say, "let us wait a little while." If you operate against his will you may subject yourself to criticism. If you lose your patient you will be blamed. It is not a matter of being convicted in the courts, but a man does not want to be harassed and criticised. He does not want public sentiment aroused against him.

Dr. Senn's book was recently introduced in court as testimony in a case in Alabama, and the surgeon who failed to use his gas test was placed before the public at a great disadvantage. Therefore a discussion of this subject by good men will materially help those who are performing these operations. If the transactions of this Association concerning such a subject are presented in court, they will have much to do toward justifying men in resorting to early operative interference. I have seen quite a number of cases, from time to time, whose lives could have been saved had they been operated upon early. If we desire to save the lives of our patients, we must operate *at once.* I have seen an extensive inflammation of the peritoneum in a great many of these cases after 12, 24 and 36 hours. I agree with Dr. Ross that it is unjustifiable for any man to go into the abdomen who has not had the benefits of experimentation on animals. He should learn how to do intestinal operations upon animals and become familiar with the *technique* before attempting to do them on human beings. Again, the surgeon should not keep the intestines out of the abdominal cavity, for the patient will die unless they are well protected. It is much better to keep them on the inside.

